# Burnout as breakdown of one’s existence in the world

**DOI:** 10.1007/s11019-025-10281-8

**Published:** 2025-07-07

**Authors:** Lisa IJzerman, Annemie Halsema

**Affiliations:** 1 Department of Philosophy, Vrije Universiteit Amsterdam, De Boelelaan 1105, 1081 HV Amsterdam, The Netherlands; 2 Department of Philosophy, Faculty of Humanities, Vrije Universiteit Amsterdam, De Boelelaan 1105, 1081 HV Amsterdam, The Netherlands

**Keywords:** Phenomenology, World alienation, Breakdown, Burnout, Merleau-Ponty, Arendt

## Abstract

Burnout is generally conceived as a condition resulting from external stressors in one’s work environment, but its precise definition is contested. In line with recent empirical studies, we suggest an existential-phenomenological approach to avoid the dualisms that characterize the present understanding of burnout. Drawing on Merleau-Ponty’s phenomenology, we do not consider burnout in terms of a psychological syndrome with physiological aspects, but rather suggest that these syndromes are expressions of the same problem. Burnout is not caused by an individual’s inability to cope with external demands, nor by a too demanding work environment, but it is a mismatch between the two. Furthermore, we conceive of ‘world’ in Arendtian terms and situate burnout within the social context of *vita activa.* We argue that burnout can be understood in terms of ‘world alienation,’ and discuss the extent to which Arendt’s diagnosis of the shifts in human activity in modernity from ‘work’ to ‘labor’ may provide a social context for the existential breakdown that burnout entails. We conclude the paper by outlining some implications for diagnosis and treatment based on our definition of burnout.

## Introduction

In ten years, between 2007 and 2017, the estimated prevalence of burnout in the general working population in the Netherlands rose from 10 to 17% (Schaufeli 2018; Houtman et al. 2019; Houtman et al. 2020). In 2019 the World Health Organization included burnout syndrome in their 11th Revision of the International Classification of Diseases (World Health Organization 2020). The universal acknowledgement of burnout as an occupational phenomenon is illustrative for the increasing attention burnout receives in modern society (Neckel and Wagner 2017). However, among scholars the concept of burnout is heavily debated, and its definition contested (Bianchi et al. 2015; Leiter and Maslach 2018; Heinemann and Heinemann 2017; Rotenstein et al. 2018; Schaufeli 2018; Nadon et al. 2022). The people suffering from burnout are the ones who pay the price as the failure to understand burnout amounts to their despair. This paper aims at formulating an answer to what we conceive of as a pressing need to better understand the phenomenon of burnout in modern society. Instead of considering burnout as a primarily psychological phenomenon, we will argue for an existential approach to it.

While the term burnout was initially primarily used for employees in the field of ‘people-work’ (Maslach et al. 2001), it later became clear that burnout occurs across a wide range of occupations, including elite sporters, volunteers and students (Bährer-Kohler 2013). Currently, it is being questioned whether burnout can be considered an exclusively work-related syndrome, or whether it also involves other straining or stressful circumstances outside of work (Schaufeli 2018; Schaufeli et al. 2020). In general, however, burnout is seen as a condition that comes forth from external stressors in one’s work environment (Demerouti et al. 2009; Schaufeli et al. 2009; Maslach and Leiter 2016; Heinemann and Heinemann 2017; Nadon et al. 2022). A recent review article describes it as “an individual response to chronic work stress that develops progressively and can eventually become chronic, causing health alterations.” (Edú-Valsania et al. 2022). In this paper, we argue that burnout is not a psychological problem within the individual caused by something external to the person—namely their work environment—but that burnout can be understood as an existential breakdown of one's relation to the world, which renders the person unable to engage with their environment in a meaningful way. This breakdown is furthermore expressed in an embodied manner, having psychological and physiological effects.

Our approach is phenomenological, in line with the two approaches of health and illness distinguished by Svenaeus (2019). Svenaeus differentiates a Merleau-Pontian approach in which one’s body when affected by illness is experienced as “conspicuous, obtrusive, or obstinate in the activities of the afflicted person” (2019, p. 461), from a Heideggerian-Gadamerian approach in which illness amounts to “finding oneself in an alienating mood that involves the whole world of the suffering person” (2019, p. 462). In this paper, we combine the two approaches to better understand the lived experience of burnout. We make use of Merleau-Ponty’s account of the body’s relation to the world, and develop the notion of ‘world’ on the basis of Arendt’s philosophy. Arendt’s phenomenological-hermeneutical analysis of the human condition can be considered as pursuing the Heideggerian-Gadamerian approach—even though her notion of ‘world’ differs from Heidegger’s in important respects.^1^ In the next section, we start with the controversies and confusions around the term burnout. In the sections thereafter we explain the existential alternative we propose.

## Burnout banter and the need for a new conceptualization

The term burnout, as used today, was coined fifty years ago by Herbert Freudenberger (1974) to refer to cases of physical or mental collapse as the result of overwork. This marked the first introduction of the term, yet states of paralyzing, mental exhaustion—the key aspect of burnout—were not new. Already in ancient Greece, a condition of world-weariness or melancholia was described. In addition, parallels have been drawn with a more recent phenomenon, namely neurasthenia—weakness of the nerves. This condition first occurred at the end of the nineteenth century and was perceived as a manifestation of modern, hectic life resulting in a cluster of symptoms such as fatigue, anxiety, headache, heart palpitations and high blood pressure, and primarily affected busy businessmen. In those days, the telephone appeared as did the steam train. It is speculated that these modern developments ultimately led people to experience extreme exhaustion which hindered them in properly functioning in daily life (Evengård et al. 1999; Schaufeli 2018).

However, quickly after the first description of modern burnout by Freudenberger (1974), Maslach and Jackson (1981) differentiated the phenomenon of burnout into three separate and measurable psychological dimensions—(1) emotional exhaustion, (2) a cynical attitude towards or detachment from work (depersonalization) and (3) a sense of professional inadequacy. These dimensions were put together in a measurement tool, the Maslach Burnout Inventory (MBI; Maslach and Jackson 1981). This definition of burnout forms the foundation of the vast majority of burnout research, and interprets it mainly as a psychological syndrome (Worley et al. 2008; Schaufeli et al. 2009; Maslach and Leiter 2016).

This psychological definition of burnout has led to several problems. Firstly, it reduces the condition to what the burnout questionnaire MBI measures (Bianchi et al. 2015; Heinemann and Heinemann 2017; Nadon et al. 2022). Several scholars have linked this circularity to a lack of reflection on—or questioning of—the underlying assumptions of burnout, as well as to a missing theoretical foundation (Schaufeli and Taris 2005; Heinemann and Heinemann 2017; Nadon et al. 2022). Secondly, ‘burnout’ at present covers the whole spectrum of symptoms ranging from mild complaints to a severe, long-term condition that significantly impairs daily functioning. What is more, burnout studies are conducted in very heterogeneous groups. This makes it impossible to compare different studies (Bährer-Kohler 2013). Furthermore, scholars differ on the causal relationships between the dimensions in the Maslach definition: some argue that emotional exhaustion leads to high levels of cynicism and depersonalization, while others suggest that exhaustion and depersonalization are the core dimensions, with professional inadequacy as a consequence (Edú-Valsania et al. 2022).

The lack of consensus in defining and classifying burnout makes it challenging to draw conclusions about its prevalence, measurement and treatment. The most reliable numbers with regard to prevalence derive from countries that have acknowledged burnout as an official diagnosis (Norlund et al. 2010; Schaufeli 2018; Houtman et al. 2019; Houtman et al. 2020). An extensive systematic review into burnout prevalence underscores the importance of developing a consensus definition (Rotenstein et al. 2018). Although a number of alternative burnout assessment methods have been developed, the MBI is still used in 88% of the burnout research publications (Schaufeli et al. 2020).^2^ As the burden of proof lies on authors to corroborate their novel perspective, it seems easier to perpetuate research that is grounded in the traditional operationalization (Nadon et al. 2022). With regard to treatment, studies investigating interventions to alleviate burnout are scarce, and findings regarding their effectiveness remain limited. For example, personal coping strategies such as mindfulness and yoga have been researched, despite evidence that social and organizational factors play a significant role in the development of burnout (Leiter and Maslach 2018). We think that addressing these concerns requires a clearer conceptual understanding of burnout.

A few attempts have been made to come up with an alternative definition of burnout. Recently Schaufeli et al. (2020) developed the Burnout Assessment Tool (BAT). This tool is based on the experiences of practitioners who deal with burnout on a daily basis, with whom semi-structured in-depth interviews were conducted. Burnout in this tool is theorized to be the combination of inability and unwillingness to spend the necessary effort at work for proper task completion (Schaufeli et al. 2020, p. 3). The definition attempts to comprehend the multi-faceted nature of burnout, bringing together the underlying condition (burnout) and its specific symptoms, and consists of four primary dimensions; exhaustion (both physical and mental), mental distance, emotional and cognitive impairment, and three secondary dimensions; depressed mood, psychological distress and psychosomatic complaints. The BAT certainly compensates for the shortcomings of earlier measurement instruments; it is more all-encompassing and covers the multi-faceted nature of burnout better than the traditional uni- or two- to three-dimensional assessment tools. Although sharing the aspiration of developing an all-encompassing conceptualization of burnout, we still have reservations about the lack of consideration of the interrelation between individual and world in the findings of Schaufeli et al. (2020).

A second alternative worth mentioning is the review article of Edú-Valsania et al. (2022) we already referred to, that takes a sociological and social-psychological approach and considers burnout as the outcome of being exposed to overly demanding or emotionally problematic working conditions. Burnout, it is argued, is to be understood mainly as motivated by organizational factors, such as work overload, lack of autonomy, role conflicts, inadequate supervision, perception of injustice, lack of social support, but it is also influenced by personality factors, such as neuroticism, emotional instability, emotion-focused coping. Also in this case, personal and organizational factors are considered as distinct. In this paper instead, we suggest an approach that considers the two as standing out to each other. What breaks down in burnout is precisely the way a person relates to and responds to their world. An approach such as that of Edú-Valsania’s, Laguía’s, and Moriano’s misses this point and does not discuss the individual’s responsiveness to their world, nor the opportunities and limitations the world offers a person.

In this paper, we pursue the phenomenological approach taken in qualitative articles that analyze the experiences of participants with burnout or exhaustion disorders (Ekstedt and Fagerberg 2005; Jingrot and Rosberg 2008; Engebretsen and Bjorbækmo 2020a, b; Finan et al. 2021). We build on their findings, including their notion of existential breakdown and their discussion in relation to Merleau-Ponty (Jingrot and Rosberg 2008; Engebretsen and Bjorbækmo 2020b), and complement these articles in proposing an alternative description of burnout. Drawing on Maurice Merleau-Ponty’s *Phenomenology of Perception* ([1945] 2014) and combining his approach with Hannah Arendt’s phenomenological analysis of human activities in *The Human Condition* ([1958] 1998), we aim at a definition of burnout, that (1) includes both its psychological and physiological symptoms and conceives of burnout as an existential problem, thereby enabling us to (2) grasp the embedding of burnout in its situational factors. We argue that burnout is not caused by *either* individual factors *or* by organizational factors, but rather by the failing interrelation between the two.

In the next section, we first of all detail the distinction between psychological and physiological symptoms that leads to misconceptions in the commonly used definitions of burnout. Then we introduce the existential approach to burnout as a breakdown between individual and world.

## Burnout as existential phenomenon

In healthcare, it is often assumed that an illness only exists when a physiological substrate can be identified (Schaufeli 2018). So far, no clear physiological substrate has been found for burnout. While some studies suggest a physiological basis—given the increased risk of cardiovascular diseases, diabetes, and respiratory infections in burnout patients (Nuallaong 2013; Salvagioni et al. 2017)—a meta-analysis of 38 biomarkers affecting various bodily systems found no specific indicators for burnout (Danhof-Pont et al. 2011). Studies with fMRI have shown reduced activity in certain brain areas, and EEG studies have detected abnormal patterns in people with burnout. However, due to the limited number of studies and uncertainty about causality, these findings remain inconclusive (Tei et al. 2014; Van Luijtelaar et al. 2010).

Despite the lack of identifiable biomarkers, burnout is associated with a wide range of physical symptoms commonly linked to chronic stress, including headaches, gastrointestinal issues, muscle tension, hypertension, sinusitis or flu-like episodes, elevated heart rate, and shortness of breath (Nuallaong 2013; Maslach and Leiter 2016; Schaufeli et al. 2020). The hegemony of the Maslach Burnout Inventory (MBI) in burnout research, which defines burnout primarily in psychological terms—such as emotional exhaustion, cynicism or detachment from work, and a sense of professional inadequacy—has reinforced the view of burnout as mainly a psychological phenomenon. At the same time, most measurement tools incorporate bodily components, such as physical exhaustion and sleep disturbances (Kristensen et al. 2005; Shirom and Melamed 2005; NVAB, LVE, and NHG 2011; Demerouti and Bakker 2008).^3^

The overall tendency is thus to reduce burnout to a mental or psychological phenomenon with possible physical consequences. Exemplary in this respect is the previously mentioned definition by Schaufeli et al. (2020), in which psychosomatic complaints are viewed as secondary symptoms. Instead of considering burnout primarily as a psychological phenomenon with physiological consequences, the earlier mentioned phenomenological studies (Ekstedt and Fagerberg 2005; Jingrot and Rosberg 2008; Engebretsen and Bjorbækmo 2020a, b) argue that burnout manifests itself often physiologically with psychological consequences as secondary symptoms.^4^ Burnout usually starts with the awareness of bodily sensations and limitations by patients. Only later feelings of anxiety or uncanniness emerge, resulting in a sense of detachment and alienation (Ekstedt and Fagerberg 2005; Jingrot and Rosberg 2008; Engebretsen and Bjorbækmo 2020a, b). Given the bodily symptoms and their relation to somatic disorders (Mohren et al. 2003; Nuallaong 2013; Maslach and Leiter 2016; Salvagioni et al. 2017; Schaufeli et al. 2020; Toppinen-Tanner et al. 2009), we suggest a phenomenological perspective that transcends the distinction between the psychological and physiological manifestation of burnout.

Instead of considering burnout in terms of *either* a physiological *or* a psychological syndrome, we consider these syndromes as expressions of the same problem. This problem is exemplified in the qualitative phenomenological studies, but it does not result in an alternative definition of burnout in these papers. Aiming to explore the lived experience of burnout, Engebretsen and Bjorbækmo (2020a) interviewed six individuals who had been on sick leave for at least three months due to fatigue‐ and pain‐related symptoms, ranging from 50 to 100%. In the development of burnout, first a phase of increasing pressure was described. Subsequently, participants mentioned that they experienced problems with achieving what they wanted. They felt more and more unable to cope, but nevertheless kept striving. They became aware of bodily symptoms and limitations, and noticed being exhausted, both physically and mentally, resulting in a psychosomatic collapse.

Burnout in these cases manifested both physically and psychologically, making it difficult to determine which came first and whether one caused the other. Similar to Merleau-Ponty’s analysis of existence in *Phenomenology of Perception* (2014), we argue that burnout cannot be understood in terms of either-or, nor simply as a combination of the two. Burnout is neither purely psychological with physiological consequences, nor purely physiological with psychological effects, and it cannot be understood as merely a combination of the two. Instead, the psychological and physiological *gear into* each other and we need to find their common ground (Merleau-Ponty 2014, pp. 80–83), namely that the individual experiencing burnout is an existential ‘being in the world.’

For Merleau-Ponty, humans are embodied sense-givers. Giving meaning to the world cannot be reduced to an activity of consciousness, but happens by means of our embodied relation to the world. The lived body thus is not a functional machine that elicits an appropriate reaction to an environmental stimulus. Merleau-Ponty breaks with the stimulus-reaction model (Reynaert 2009). ‘Being in the world’ for Merleau-Ponty refers to pre-objective standing out to the world that cannot be reduced to third person processes or to first person experiences. Instead, it aims at our embodied existence in which the body is at once the vehicle for having a world (Merleau-Ponty 2014, p. 147) and united with a definite milieu in which it is engaged (2014, 84). Carel elucidates that in a normal situation, the body-subject engages in a ‘primordial dialogue’ with the world. “This dialogue is a pre-reflective absorbed engagement with the environment, and takes place constantly in everyday activities.” (Carel 2016, p. 30).

In the case of burnout, the normal way of being in the world is broken. Engebretsen and Bjorbækmo also signal this: “The way the participants previously experienced themselves as being in the world felt altered and obstructed.” (2020a, p. 444) The person experiencing burnout is an embodied subject situated in the world, that has lost contact with their world. A breakdown happens between the individual and their environment that cannot be explained as mainly physiological or mainly psychological, but that takes place at the existential level that underlies the ways burnout manifests itself. Merleau-Ponty explains this level as follows: “Thus, our ‘world’ has a particular consistency […] that forbids treating ‘being in the world’ as a sum of reflexes, and the pulsation of existence has a particular energy […] that precludes treating it as an act of consciousness. Because it is a pre-objective perspective, being in the world can be distinguished from every third person process, from every modality of the *res extensa*, as well as from every cogitatio, from every first person form of knowledge—and this is why ‘being in the world’ will be able to establish the junction of the ‘psychical’ and the ‘physiological.” (Merleau-Ponty 2014, p. 82). Hence, ‘being in the world’, or existence forms the common ground and it is this underlying ‘pre-objective perspective’ that has been altered in burnout, with physiological and/or psychological syndromes as the result. These syndromes manifest burnout and can be conceived of as expressions of the breakdown of the existential relation with the world.

### ‘I cannot’

The incapacity to act in the world that characterizes burnout thus cannot be explained in terms of an individual’s *unwillingness* to engage with the world. Individuals suffering from burnout experience emotional exhaustion, cynicism, and a sense of professional inefficacy (Maslach and Leiter 2016), as mentioned before. Feelings of failing to achieve goals and of alienation, as well as a psychosomatic collapse or breakdown are described (Ekstedt and Fagerberg 2005; Jingrot and Rosberg 2008; Arman et al. 2010; Engebretsen and Bjorbækmo 2020a, b). These symptoms impede normal functioning in a person’s occupational and/or social life. This is not a voluntary act of the person who suffers from burnout. Instead, this person is *unable* to act: their body is no longer open to the possibilities of the world. Engebretsen and Bjorbækmo describe this phenomenon as follows: the “sufferer from burnout comes face-to-face with the radical contingency of his or her existence and the inescapable limitations of their embodiment” (2020b, p. 1474). Burnout, in other words, can be described in terms of the individual’s embodied ‘I cannot.’ Merleau-Ponty famously suggests that consciousness is embodied and argues that it is originally “not an ‘I think that’ but rather an ‘I can’” (2014, p. 139). Underlying the Cartesian notion of consciousness as ‘I think’ is this original relation between our body and the world, in which we experience this relation in terms of being-able-to.

Jenny Slatman elucidates that ‘I can’ does not so much refer to physiological or psychological impossibilities, but rather to the potentiality of relating to and dealing with the world and the situation in which one lives (2023, p. 69). In burnout this potentiality, in other words the field of possibilities of the individual, is limited. The world one used to have before developing burnout becomes narrower. The ‘I can’ is impaired and transforms into an ‘I cannot.’

Burnout as a breakdown of one’s existence in the world therefore has two different facets. It refers to the existential level of a person’s being in the world, in which the psychological and physiological gear into each other. Burnout may manifest itself physiologically or psychologically, and most often both. All of these symptoms are expressions of the problem of not being able to relate to one’s world. The second facet addresses the interrelation between individual and world. Burnout implies a breakdown in this relation. The individual feels distanced from their world, not only from their work environment, but burnout also has repercussions for one’s social life (family and friends). To conclude, from a Merleau-Pontian perspective, we can explain the changes in the experience of the world that result from embodied consciousness. But what does the still abstract notion of ‘world’ imply, that in burnout is at a distance? In order to understand how burnout is embedded in its environmental factors, we must further delve into this world.

## The world as a source for burnout

People suffering from burnout mention that their experiences primarily center around'doing' rather than'being' (Ekstedt and Fagerberg 2005). They sense an incapacity to act, which we just considered in terms of ‘I cannot’ in relation to the standards of their work and private environment. In Merleau-Ponty’s phenomenological account of a breakdown of one’s existence, the subject stands out to the world and is in interrelation with it, but the demands this world places on the individual are not accounted for in his philosophy. For this purpose, we turn in this section to Arendt’s *The Human Condition* (1998), who famously discusses active life, *vita activa*, or, as she herself phrases it: “what we are doing” (Arendt 1998, p. 5). We argue that burnout is not simply related to ‘I cannot’ on an individual level, but that it is for a large part associated with the predominant ways in which individual and world interrelate, that is, by the societal way *vita activa* is given form. This implies, as we will contend in this section, that the exhaustion characteristic of burnout is not only existential in nature but also a signal of the alienation that characterizes modern work relations.

Relating Arendt’s political theory of *vita activa* and world to Merleau-Ponty’s notion of the existential relation between subject and world, for a novel definition of burnout, might seem eclectic. Yet, Arendt’s work has been described as a “phenomenology of the human condition” (Vasterling 2011, p. 82). She famously describes the different types of human activity through which we constitute our world, namely (1) labor, (2) work and (3) action*.* While ‘labor’ refers to the repetitive reproduction of life (e.g., food production, care for the body); ‘work’ involves the creation of artificial things, both material and immaterial, that outlive us (ranging from a wooden chair to books) (Arendt 1998, p. 7). ‘Work’ implies the creation of permanent objects around us that foster familiarity with the world, as it is partly of our own making. The notion ‘action’ revolves around the establishment of a common world through human plurality, it refers to the intersubjectively constituted network of action and speech through which we articulate what the world is for us (Arendt 1998, p. 7). The distinction between these activities, along with Arendt’s diagnosis of the shifts in *vita activa* in modernity, will be of use for our analysis of burnout. But first, we specify the difficulties of combining a Merleau-Pontian and Arendtian approach in conceiving of burnout.

In the existential description outlined above, we explained that burnout may manifest itself physiologically or psychologically and that these realms gear into each other. An important problem signaled in Arendt scholarship is, however, that she conceives of the body as “threat to human plurality” (Zerilli 1995, p. 171). For Arendt, maintenance of the body is part of the private sphere, and human plurality is expressed by means of speech and action and not through the body (Arendt 1998, p. 179). In contrast, we have seen that, for Merleau-Ponty, we are always already bodily engaged in all our activities. Does this distinction between the two philosophers undermine our argument? We argue it does not.

First of all, several scholars have challenged and specified Arendt’s claim regarding her resentment of the body (Schoonheim 2019; Portes 2021). More importantly, however, the activities we are primarily interested in—work and labor—clearly engage the body. Labor for Arendt is the activity “which corresponds to the biological process of the human body” (1998, p. 7). It entails the sphere of growth and decay of the body, and is not so much about the creation of finished products, but about serving the needs for the maintenance of life (1998, p. 83). By means of work, we use our body to produce things, we create a “man-made world of things” (1998, p. 173)*,* that outlasts us*.* Examples are working with wood and stone (1998, p. 136, n.1), in which the fabricator ‘works upon’ material. Yet, Arendt also has an open eye and provides examples of activities (“tilling the soil,” the problem of use) in which labor and work are closely related. The thesis that Arendt ignores the body, which makes it questionable to bring her philosophy into relation with Merleau-Ponty’s, thus needs to be nuanced.

Returning to our topic, to what extent is ‘the world’ a source for burnout? In the next section, we relate the existential breakdown that typifies burnout with Arendt’s ‘world alienation,’ which includes first of all an alienation from the products that we create and secondly from other people and the self.

### World alienation

Hannah Arendt in *The Human Condition* (1998) analyzes shifts in *vita activa* over 21 centuries, between antiquity and modern society, and within modernity from *homo faber* to *animal laborans*. Burnout, however, was introduced as a notion to refer to physiological and mental exhaustion only fifty years ago.^5^ To what extent can Arendt’s diagnosis thus be used to explain the phenomenon of burnout? In this section, we discuss the relationship between the symptoms of burnout and her analysis of the shift in types of human activity—specifically, the rise of labor in modern society—and the resulting world alienation. This will help us understand how to situate burnout within its societal context and the core human activities that shape it.

Arendt argues that in modernity a shift occurs in human active life from *homo faber* to *animal laborans*. She signals that, compared to antiquity, the central human activity in present-day consumer society has shifted to labor. *Animal rationale* has been replaced by *animal laborans* (1998, 84). This shift means that the realm of necessity, which she distinguishes from the realm of freedom—the political sphere—has now entered the public sphere. In antiquity, labor was the work of slaves in the household, who tended to the necessities required to reproduce life (1998,pp. 83–84). However, in modern times, it has become the core human activity.

Arendt writes that the industrial revolution "has replaced all workmanship with labor, and the result has been that the things of the modern world have become labor products whose natural fate is to be consumed, instead of work products which are there to be used" (1998, p. 124). The permanence, durability and stability of work have been “sacrificed to abundance” (1998, p. 126), which can only be brought about by laboring. This shift implies that the objects produced have become consumer products (1998, p. 124). Work has been fragmented into its smallest components, allowing for division of labor and ensuring both the necessities of life and their abundance (1998, p. 126). The durable products of ‘work,’ (Arendt names chairs and tables), have been reduced to disposable objects, quickly used up like dresses or food. Instead of following the Marxian analysis of self-alienation, Arendt argues that the consequence of this domination of labor in the contemporary world is ‘world alienation’ (1998, pp. 248–257). Whereas Marx saw alienation in the laborer’s estrangement from the products of their labor (1961), Arendt sees a broader loss: the eclipse of a common public world (1998, p. 257) and the emergence of a worldless mentality in modern man.

In discussing world alienation, Arendt notices a desire to escape the company of others, to reject the common world. In modern society, people prefer to focus on themselves and take a flight “from the world into the self” (Arendt 1998, p. 6), rather than focus on others and the community they live in. Thus, world alienation has different aspects: 1. Losing a sense of the world we commonly create, both material and intangible; 2. Feeling alienated from others in one’s environment; and ultimately 3. Alienation from the self—as it is life with others that makes life worthwhile and human. This tendency aligns with how modern society is often classified as an individualized society, where personal choice prevails over collective action and social communities (Rasborg 2017; Verhaeghe 2018).

Arendt’s notion of world alienation helps us understand how these developments contextualize the phenomenon of burnout. The loss of connection to a shared world and the retreat into the self resonate strongly with the experience of burnout. Firstly, this is reflected in the dominant definition of burnout, as the key concepts are formed by feelings of cynicism, mental distance, depersonalization and disengagement (Maslach and Leiter 2016). Secondly, burnout sufferers themselves report symptoms of alienation. In qualitative research, feelings of alienation and detachment from the world, from themselves and others are commonly mentioned (Ekstedt and Fagerberg 2005; Jingrot and Rosberg 2008; Engebretsen and Bjorbækmo 2020b).^6^ In line with this, a loss of homelikeness in the body and the familiar world have been described (Heidegger [1927] 1962; Jingrot and Rosberg 2008). Lastly, it was found that burnout sufferers express a lack of community and that those who experience support in their surroundings are less likely to develop burnout (Halbesleben and Buckley 2006; Listopad et al. 2021).

Thus, Arendt’s observation of the shifts in *vita activa*, along with the different aspects of world alienation, provide a valuable perspective on the phenomenon of burnout situating it in a socio-historical framework. In the next section, we will further analyze how Arendt’s theory helps contextualize ‘world’ within the existential breakdown of burnout.

### Work and labor

Despite the lack of a precise temporal overlap between the rise of labor and the emergence of burnout as a modern phenomenon, burnout can be interpreted through the lens of Arendt’s conception of ‘world’. In this section, we further explore Arendt’s account of the replacement of ‘work’ and ‘action’ by ‘labor’ in modern society and consider what this shift might reveal about our understanding of burnout*.* We argue that while the creation of durable products—typical of ‘work’—is considered as protection against burnout, it cannot be regarded as a remedy that reliably prevents it. The reason is that many of those who suffer from burnout (such as teachers, healthcare workers; see APA 2022) are engaged in activities that fall within Arendt’s category of ‘work’. Moreover, Arendt’s devaluation of the activities that she classifies as ‘labor’ is not supported by studies on burnout prevention.

Several studies argue that the creation of things that outlast us—which for Arendt is typical for the activity of work—could protect against burnout (Hadi et al. 2021,7; Jingrot and Rosberg 2008; Viding et al. 2015). For instance, it was found that white-collar workers more often experience stress-related disorders than blue-collar workers (Jingrot and Rosberg 2008). Another study found that cultural activities such as drawing, painting and interactive theater had a positive effect on exhaustion and self-rated health (Viding et al. 2015).^8^ Creating endurable products, hence working, might decrease existential exhaustion for the following reasons. Firstly, work is associated with feelings such as pride of what you produce, self-confidence, satisfaction, and with existential necessities that it brings forth—immortality, familiarity, stability (Arendt 1998; Sennett 2008). Secondly, work anchors people in tangible reality (Sennett 2008). That is, one meets nature in working, one transforms the materials available in order to create something new. Thirdly, engagement with the things you produce and with others (Edú-Valsania et al. 2022), is to be found in working.

In line with Arendt’s diagnosis of the shift from work to labor, American sociologist and former student of Arendt, Richard Sennett (2008) claims that in late capitalism we have lost the craftsmanship’s abilities. The creation of human products by the activity of work in present-day capitalist society has collapsed. As time pressure reigns in late modernity, there is no room for people to create good quality, long lasting products to which they can dedicate themselves. There is no time for the craftsman, an engaged human being, completely absorbed in practical activity (Sennett 2008, p. 20).^9^ It is no longer profitable to make artifacts that endure. It is more cost-effective to produce things that can be quickly replaced or that will soon be out of fashion (Gómez 2016). In addition, late modern society is marked by an overall loss of appreciation of manual labor. Nowadays, ‘brain’ work is highly esteemed yet working with one’s hands is seen as worthless (Sandel 2020, pp. 267–268).

Yet, it is not only people doing repetitive labor who burn out. Instead, the majority of burnout sufferers are for instance teachers and healthcare workers (APA 2022). In their jobs, they engage with others, and although they may not create tangible products, their work does have a lasting influence on others and falls into the category of intangible products that Arendt mentions in the context of work (such as words, 1998, p. 19). However, this is not a counterargument to our claim that the shift from work to labor in late modernity provides a context for the phenomenon of burnout. That is, the reasons for burnout mentioned in several studies do not so much relate to the *content* of the job or what is produced, but rather to the *circumstances* in which it takes place (Demarzo et al. 2020; Edú-Valsania et al. 2022). Reasons for burnout are in the first place work overload and secondly emotional labor.^10^ Other reasons that are mentioned are lack of autonomy or influence at work, not knowing what is expected of them or incongruent demands, inadequate supervision, lack of social support, poor working hours. This implies that the reasons why people burn out—even if their jobs fall into the Arendtian category of ‘work’—do not solely relate to the content of their tasks, but also involve the context in which they must fulfill these tasks.

A case in point is the increasing pressure on healthcare workers due to high work demands (Rink et al. 2023). This might transform their work into ‘labor,’ i.e., a cyclical execution of caring duties to be conducted as quickly as possible, moving from one patient to the next, rather than having the time to make a lasting, personal impact and to perform their work as actual'work.' Additionally, systems-level issues (such as technology and administrative tasks) might prevent healthcare workers from fulfilling their primary job (patient care) (Rink et al. 2023), just as teachers experience stress due to, among other factors, shifting educational policies and disengaged or dissatisfied parents (Herman et al. 2020; Anton and Van Ryzin 2024). These circumstances in which people need to do their job reinforce our argument for considering burnout within the context of the shifts in human activities that Arendt signals.

While we thus hold on to the thesis that the shift from work to labor enforces burnout, we still need to take a distance from Arendt’s depreciation of labor. Labor’s very rhythm, its cyclical nature (which characterizes daily activities such as baking bread, gardening, doing the dishes, in other words, caring for one’s own and others’ bodies), is considered preventive of burnout (Bernhardsson et al. 2016; Edú-Valsania et al. 2022). The overlap with work in these cases is that the actions have a clear end result and purpose. Additionally, as many laborers in modern society do not suffer from burnout, the same argument made about'work' could apply to'labor'; the causes of burnout lie not merely in the job's content or its output, but in the conditions under which it is performed.

Even though not all aspects of Arendt’s diagnosis of modernity can be related to burnout, we think that her definition of world alienation and the importance she grants to ‘work’ help in understanding this phenomenon. The existential breakdown between individual and world that characterizes burnout is to be situated within late capitalism’s consumer society.

## Consequences for the treatment of burnout

In this paper, we aimed at a better understanding of burnout, for the sake of those suffering from this ill-understood phenomenon and the healthcare practitioners who diagnose and treat them. We proposed an existential-phenomenological Merleau-Pontian approach in which the psychological and physiological symptoms are seen as resulting from the breakdown of the relation between the individual and their world. Burnout should, first of all, be considered as ‘I cannot,’ that is, as a mismatch between the patient and their work and social environment. Next, using Arendt’s framework, we contextualized this breakdown and situated it in the present-day consumer society. As we argued, burnout can be understood in terms of ‘world alienation,’ referring to the distance it forms between the individual and the world shared with others, ultimately leading to self-alienation.

The bodily symptoms of burnout, such as physical exhaustion, headaches and sleep disturbances, along with the psychological feelings of anxiety and detachment found in empirical phenomenological studies, gain a broader perspective by considering burnout in terms of an existential breakdown in the relation between individual and world. But what does this phenomenological understanding of burnout contribute to healthcare practices where patients with burnout symptoms are treated? We conclude the paper by detailing a few implications of our new definition for the diagnosis and treatment of burnout.

First of all, as long as burnout does not have a clear demarcated biological substrate, it is considered a psychological problem. This may be confusing for patients as they also experience physiological problems. In our alternative description, burnout is not a psychological problem, but an existential one that may manifest itself physiologically and/or psychologically. This description may give burnout sufferers a better understanding of their problems and incite acceptance of the problems of burnout sufferers by healthcare practitioners.

Contextualizing the symptoms of burnout may help individuals in overseeing the reasons for their suffering. Depending on the severity of their burnout, they may need assistance with time management, job crafting, or even exploring a new direction in their professional life.^11^ In psychoeducation, healthcare practitioners can inform patients about the causes of burnout and contextualize it without explicitly emphasizing the psychological and individual nature of burnout, as it is typically understood. This approach could help identify solutions that are more suited to the patient’s specific situation.

Another consequence for healthcare is that practitioners will need to be informed about the patient’s situation. Merely focusing on symptoms is not sufficient; practitioners must also understand the patient’s work environment and social context. In this respect, a narrative approach (Charon 2006), which allows space for the patient’s life story, is more informative than a questionnaire. We realize that a narrative approach may be time-consuming and perhaps not feasible in present-day healthcare. As healthcare providers are among those who suffer most from burnout, it would be too much to ask them to engage in detailed, therapy-style investigations into the patient’s situation. Practitioners lack the time and training; therefore we recommend considering referring the patient to occupational therapists, social workers or primary care mental health practitioners, to address the larger picture.

Lastly, we suggest that future research should further explore the experiences of those suffering from burnout, specifically investigating what they perceive as most crucial for understanding their condition and which aspects of their healthcare practitioner's attitude or explanation they found helpful. How would they like to be understood? Conducting further research based on the experiences of burnout sufferers might reveal implicit assumptions in traditional medicine that are not beneficial to the patients themselves. Additionally, it could be examined to what extent the Arendtian concepts of ‘work’ and ‘labor’ play a role in one’s job and how working conditions influence them. Could an Arendtian perspective on burnout help in reorienting after an existential breakdown? Also, the existential construct of temporality may be relevant to a more comprehensive understanding of burnout. Merleau-Ponty elucidates that we retain traces of our past, both psychic and physiological, while "my world is carried along by intentional lines that trace out in advance at least the style of what is about to arrive" (2014, p. 439). In line with this, lived experiences of individuals suffering from burnout reveal that "the significance of past, present, and future is changed," and that their intentional direction appeared to be pointing ‘backwards’ in time rather than ‘forwards’, which may be significant for the process of recovery (Engebretsen & Bjorbækmo 2020b, p. 1475). Future research could therefore further explore the existential dimension of temporality in relation to burnout, as it may enrich our understanding of its experiential and existential depth.

We hope that with our comprehensive definition of burnout as breakdown of one’s existence, those suffering from it can better understand themselves and be better understood by those around them, their healthcare practitioners, and the burnout scholarly field.

## Footnotes

1 ‘World’ for Arendt pertains to the human-made world that she distinguishes from the earth we are thrown on to. Also, ‘thrown’ for her has another meaning than it has for Heidegger; for Arendt it pertains to being born. (The Hannah Arendt Center for Politics and Humanity https://hac.bard.edu/amor-mundi/guided-into-the-world-2013-02-04).

2 Several other measurement instruments have been developed, including The Shirom-Melamed Burnout Questionnaire (SMBQ; Shirom and Melamed 2005), which makes a distinction between the physical, emotional and cognitive features of exhaustion and the Copenhagen Burnout Inventory (CBI; Kristensen et al. 2005) which evaluates both physical and psychological exhaustion. There also is the OLdenburg Burnout Inventory (OLBI), which is founded upon a two-dimensional construct and consists of both exhaustion– emotional, physical and cognitive components– and disengagement (from work) (Demerouti and Bakker 2008). More recently, Schaufeli, Desart, and De Witte (2020) developed a new measure of burnout, which relies on an improved definition of burnout. Already, emerging evidence supports the psychometric properties of the scores (Nadon, De Beer, and Morin 2022). However, it turns out to be difficult to reach consensus on a new definition, as aforementioned endeavors remain counter to the prevailing method in burnout research, which still draws on the MBI (Nadon, De Beer, and Morin 2022).

3 In addition, the influence of physical activity on burnout has been researched (Dreher, Dößereck, and Lachtermann 2020; Naczenski et al. 2017).

4 For instance, Jingrot and Rosberg (2008) interviewed eleven participants on sick leave due to exhaustion disorder. The participants describe how they became aware of bodily sensations and symptoms such as chest pain, sinusitis, fatigue and muscular pain, which lead to anxiety and ultimately to a sense of detachment from the world.

5 As previously mentioned, states of excessive exhaustion were described before the introduction of the term burnout. However, in this article we analyze burnout as the modern phenomenon first described in 1974 and developed since, placing it in its contemporary context, since it is this recent phenomenon that is increasingly discussed today.

6 Note that a sense of depersonalization has also been touched upon by Merleau-Ponty in his discussion of consciousness and mental illness (2014, 138–139).

7 This also goes for unpaid forms of craftsmanship. This study was conducted to investigate whether leisure crafting could decrease emotional exhaustion indeed found this correlation. It was carried out in the context of the COVID-19 pandemic and concluded that crafting made reduction in emotional exhaustion possible through the creation of meaningful leisure experiences (Hadi et al. 2021). Although the concrete activities of “leisure crafting” are not specified, the authors mention it is related to setting and reaching goals, learning and personal development.

8 In this study, the participants suffered from burnout symptoms and engaged in a cultural activity course that took place every week for 3 months.

9 Note that engagement has been taken up by various scholars as a possible antithesis of burnout, as a potential protective factor for burnout. This assumption is still up for debate, since the exact description of engagement is yet to be defined, however consensus exists on its foundation, namely a productive and fulfilling state within one’s working environment (Maslach and Leiter 2016).

10 Emotional labor is defined as follows: “the psychological process necessary to self-regulate one’s emotions and show those emotions desired by the organization” (Edú-Valsania, Laguía, and Moriano 2022).

11 This is in line with a phase of reorientation that has been described in qualitative research as a follow-up for the psychosomatic collapse that marks burnout (Ekstedt and Fagerberg 2005; Jingrot and Rosberg 2008; Arman et al. 2010; Engebretsen and Bjorbækmo 2020a, b). Our definition of burnout as a disruption of one’s existence explains that one has to subsequently reorient themselves in the world.
